# Latent toxoplasmosis and preeclampsia: evidence for the role of chronic infection in pregnancy complications

**DOI:** 10.1186/s12884-025-08052-7

**Published:** 2025-10-24

**Authors:** Seyed Abdollah Hosseini, Bahareh Basirpour, Seyedeh Kiana Emadi Jamali, Mitra Sadeghi, Bahman Rahimi Esboei, Shahabeddin Sarvi, Marzieh Zamaniyan, Ahmad Daryani, Sargis A. Aghayan

**Affiliations:** 1 Department of Parasitology and Mycology, faculty of Medicine, Mazandaran University of Medical Science, Sari, Iran; 2 Toxoplasmosis Research Center, Communicable Diseases Institute, Mazandaran University of Medical Sciences, Sari, Iran; 3 Student Research Committee, School of Medicine, Mazandaran University of Medical Sciences, Sari, Iran; 4 Diabetes Research Center, Mazandaran University of Medical Sciences, Sari, Iran; 5 Sexual and Reproductive Health Research Center, Mazandaran university of Medical Sciences, Sari, Iran; 6 Laboratory of Molecular Parasitology, Scientific Center of Zoology and Hydroecology, NASRA, 7P. Sevak St, Yerevan, 0014 Armenia; 7 Laboratory of Zoology, Research Institute of Biology, Yerevan State University, 1 Alex Manoogian, Yerevan, 0025 Republic of Armenia; 8 Department of Obstetrics and Gynecology, School of Medicine, Diabetes Research Center, Sari Imam Khomeini Hospital, Mazandaran University of Medical Sciences, Sari, Iran

**Keywords:** *Toxoplasma gondii*, Seroprevalence, Preeclampsia, Pregnancy complications, Risk factors

## Abstract

**Background:**

Preeclampsia (PE) is a major global health concern, contributing to adverse maternal and perinatal outcomes. While its exact pathogenesis remains unclear, abnormal placentation, immune dysregulation, and systemic inflammation are implicated. Emerging evidence suggests that chronic infections, including toxoplasmosis may increase PE risk. This study investigates the seroprevalence of *Toxoplasma gondii (T. gondii)* infection in pregnant women with and without PE and analyses associated risk factors.

**Methods:**

This comparative study examined 180 participants (90 preeclamptic cases and 90 healthy controls) from prenatal care centers in Mazandaran, northern Iran. ELISA testing measured *T. gondii* IgG and IgM antibodies levels in collected serum specimens.Thepotential risk factors through validated surveys capturing demographic characteristics, nutritional patterns, educational background, and toxoplasmosis knowledge were assessed. The investigation employed a matched case-control design to evaluate associations between seropositivity and clinical outcomes.

**Results:**

The seroprevalence of anti-*T. gondii* IgG was significantly higher in the PE group (71.11%) compared to controls (56.66%) (*P* = 0.045), while no IgM-positive cases were detected, suggesting chronic infection. Logistic regression analysis identified age > 30 years, consumption of undercooked meat, lower educational attainment, and lack of awareness regarding toxoplasmosis as independent risk factors for toxoplasmosis-associated PE.

**Conclusion:**

The findings reveal that chronic toxoplasmosis is associated with an increased risk of PE, particularly among older housewives and women with limited health literacy. Public health interventions, including education on safe food handling practices and prenatal screening, have the potential to reduce both infection rates and the incidence of PE.

## Introduction

Preeclampsia (PE) characterized by new-onset hypertension and proteinuria after 20 weeks of gestation is a major global health concern in obstetrics, significantly contributing to maternal and neonatal morbidity and mortality [1, 2]. Based on the World Health Organization (WHO) reports, preeclampsia affects between 2% and 5% of pregnancies worldwide [3]. Moreover, annually about 46,000 maternal deaths and 500,000 fetal or newborn deaths are reported due to the preeclampsia [4].

Current hypotheses emphasize multifactorial origins, including abnormal placentation, immune dysregulation, cardiovascular maladaptation, and systemic inflammation [5, 6]. PE is associated with a shift from a Th2 to a Th1 cytokine profile, marked by elevated levels of pro-inflammatory cytokines such as TNF-α, IL-12, and IFN-γ [7, 8]. Emerging research suggests infections may play a role in both the onset and progression of PE, potentially through mechanisms involving acute uteroplacental atherosis and intensified maternal systemic inflammation [9, 10]. Epidemiological data support an association between maternal infections (bacterial, viral, and parasitic) and increased PE risk [11].

Among parasitic agents, protozoans such as *Toxoplasma gondii* (*T. gondii*), have garnered attention due to their impact on maternal and fetal outcomes [9, 12]. *T. gondii* is an intracellular protozoan parasite that causes toxoplasmosis and affects over 30% of the global population [13]. This parasite is transmitted through ingestion of contaminated meat, oocysts, congenital transmission, blood transfusion, and organ transplantation [14, 15]. Furthermore, *T. gondii* may induce immune responses and causes elevated levels of pro-inflammatory cytokines such as TNF-α, IL-6, and IL-12 [16] that are central to the inflammation condition that implicated in PE pathogenesis, contributing to endothelial dysfunction, increased vascular resistance, and placental insufficiency hallmark features of the disease [17]. Moreover, *T. gondii* infection during pregnancy is linked to complications such as low birth weight, chorioretinitis, fetal abortion, and congenital anomalies, and may also contribute to psychiatric conditions [18, 19].

Based on this evidence, it is hypothesized that *T. gondii* infection may increase the risk of PE through mechanisms involving systemic inflammation or utero-placental atherosis. Considering the significant clinical and public health implications of PE, alongside the widespread prevalence of *T. gondii* infection, further investigation is warranted. Accordingly, this case-control study was designed to test this hypothesis and to evaluate the potential association between *T. gondii* infection and PE among pregnant women in northern Iran, a region endemic for toxoplasmosis.

## Materials and methods

### Ethical approval

The study protocol was approved by the Institutional Ethics Committee of Mazandaran University of Medical Sciences (Approval No. IR.MAZUMS.REC.1402.17224). Written informed consent was obtained from all participants before sample collection. The study adhered to the ethical principles outlined in the Declaration of Helsinki (2013 revision). To ensure participant confidentiality and privacy, all collected data were anonymized and securely stored.

### Study design and study populations

A retrospective case-control investigation was performed in Mazandaran Province, Iran, in 2022–2023. Using a random cluster sampling and based on the inclusion and exclusion criteria, 90 pregnant women diagnosed with PE as the case group and 90 normotensive pregnant women as the controls who has referred to obstetrics and gynecology departments in hospitals and treatment centers in Mazandaran province were included in this study.

Participants provided written informed consent before enrollment. A standardized questionnaire, administered by a trained clinician, collected demographic information including place of residence, cat exposure residence, occupation, meat consumption, education and awareness of *T. gondii* as well as medical and obestric date including maternal age, trimester of pregnancy and miscarriage history.

The inclusion criteria including: (1) Pregnant women after week 20 of pregnancy that present mild gestational hypertension confirmed by at least two blood pressure (BP) measurement ≥ 140/90 mmHg with an interval of at last 4 h; (2) Proteinuria ≥ 300 mg/day in a 24-hour urine sample or the protein to creatinine ratio ≥ 30 mg/mmol in a random urine sample; (3) Women with HELLP (Hemolysis, Elevated Liver enzymes, and Low Platelet count) syndrome, Idiopathic cephalalgia (treatment-resistant headaches without alternative etiology); (4) Ophthalmologic manifestations including visual field abnormalities and photopsia; (5) New-onset, refractory headache that was not be diagnosed otherwise and does not respond to usual doses of painkillers.

Exclusion criteria including: (1) Advanced maternal age (≥ 45 years); (2) Pregnancies achieved through assisted reproductive technologies (including in vitro fertilization or oocytes donation); (3) Active substance use disorders; (4) Patient with chronic hypertension diagnosed before pregnancy; (5) Women with CMV, HSV, HIV/AIDS, autoimmune diseases, or gestational diabetes.

Clinical parameters collected from both case and control groups encompassed demographic and obstetric variables such as maternal age, gravidity, parity, mode of delivery (vaginal or cesarean), pregnancy loss history (spontaneous abortions or stillbirths), gestational age at enrollment, prior preeclampsia occurrences, and hemodynamic measurements (systolic/diastolic blood pressure and mean arterial pressure).

### Measurement of anti-*T. gondii* IgG and IgM antibodies

Five mL venous blood samples from each participant were obtained and centrifuged to separate serum, which was subsequently stored at − 20 °C for analysis. IgG and IgM antibodies specific to *T. gondii* were measured in all samples by a commercial ELISA (Enzyme-Linked Immunosorbent Assay) kit (Pishtaz Teb, Iran; 100% Sensitivity, 99% specificity for anti-*Toxoplasma* IgM detection, 100% sensitivity and 100% specificity for anti-*Toxoplasma* IgG detection) following the manufacturer’s standard procedure with seropositivity considered at a cut-off of ≥ 1.1 IU/mL.

### Statistical analysis

For calculation of the sample size, we used a 95% confidence interval (CI) level, a power of 80%, a 1:2 proportion of cases and controls, a reference seroprevalence of 58.8% [14] as the expected frequency of exposure in controls, and an odds ratio (OR) of 2.33. The result of the sample size calculation was 90 cases and controls. The results of serology tests and the raw data of the questionnaires were analyzed by SPSS version 23 statistical software using descriptive statistics, Chi-square test, odds ratio with a 95% confidence interval. Among the variables that showed a significant association with toxoplasmosis, a multivariate logistic regression analysis was performed to adjust for potential confounders. The P-value < 0.05 was considered statistically significant.

## Results

### Seroprevalence of *T. gondii* infection

A seroprevalence comparison between healthy pregnant women and those with preeclampsia revealed that anti-*T. gondii* IgG antibodies were detected in 56.66% (51/90) of the control group compared to 71.11% (64/90) of the PE group. Statistical analysis demonstrated a significant difference in seropositivity between the two groups (*p* < 0.05). Specifically, pregnant women with latent toxoplasmosis (IgG-positive) exhibited 1.88 times higher odds of developingPEp than their seronegative counterparts (OR = 1.88, 95% CI: [1.01–3.55], *p* = 0.045).

### Seroprevalence of *T. gondii* infection based on the demographic characteristics

The study population comprised 90 preeclamptic women (cases) and 90 normotensive pregnant women (controls), matched for key demographic variables (Table 1). Age distribution revealed 34 (37.8%) cases and 31 (34.4%) controls were < 30 years, while 56 (62.2%) cases and 59 (65.6%) controls were ≥ 30 years. Age-Stratified Analysis demonstrated significantly higher *T. gondii* IgG seropositivity among PE women ≥ 30 years (80.36%) compared to younger cases (55.88%; *P* = 0.013). While controls showed a similar age-related trend (61.02% vs. 48.39%), this difference was not statistically significant (*P* = 0.25). In the case group, women ≥ 30 years showed 2.61-fold higher seroprevalence (OR = 2.61, *P* = 0.024). PE group exhibited higher *T. gondii* seroprevalence among patients with cat exposure history (83.33% vs. 69.23%; *P* = 0.31). Controls showed significant elevation (81.25% vs. 51.35%; *P* = 0.05). Intergroup comparison revealed non-significant difference (OR = 1.15, *P* = 0.91). Housewives predominated in both groups (cases: 62/90; controls: 71/90). Significant occupational association emerged among cases (housewives 85.48%; *P* < 0.001) but not controls (60.56%; *P* = 0.33). Among PE women, undercooked meat consumption correlated with higher seroprevalence (85.29% vs. cooked meat consumers; *P* = 0.02). Furthermore, consumption of undercooked meat elevated the *T. gondii* seroprevalence 2.04 times (*P* = 0.19). Significantly elevated seroprevalence occurred among cases with academic education (OR = 2.32, *P* = 0.039) versus controls. University-educated participants showed non-significant elevation (OR = 2.15, *P* = 0.21). The results of the current study indicated that the seroprevalence of anti-*T. gondii* IgG antibodies among PE women in their second and third semester of pregnancy (59.26% vs. 76.19%, *P* = 0.12) was not significantly different, but the seroprevalence of anti-*T. gondii* IgG antibodies among PE women (76.19%) was higher than in control (OR: 2.24, *P* = 0.06). Moreover, the prevalence of anti-*T. gondii* IgG antibodies was higher in PE women with history of miscarriage (63.64%) in comparison with control but statistical analysis did not show any significant difference (OR = 1.12, *P* = 0.67).

**Table 1:** Statistical analysis of various factors associated to seropositivity for *Toxoplasma gondii* among pregnant women with preeclampsia and comparison with a healthy group in Northern Iran

Variables		Pregnant Women with Preeclampsia (*n* = 90)				Healthy Pregnant Women (*n* = 90)				Odds ratio (CI 95%)	*P*- value
		No.	Positive	percentage	*P*- value	N0.	Positive	Percentage	*P*- value		
Maternal Age	< 30	34	19	55.88%	0.013	31	15	48.39%	0.25	1.35 (0.45–4.01)	0.62
	> 30	56	45	80.36%		59	36	61.02%		2.61 (1.12–6.21)	0.02
Place of residence	City	38	24	63.16%	0.15	43	21	48.84%	0.15	1.79 (0.73–4.43)	0.21
	Rural	52	40	76.92%		47	30	63.83%		1.89 (0.78–4.62)	0.16
Cat exposure history	Yes	12	10	83.33%	0.31	16	13	81.25%	0.05	1.15 (0.14–11.23)	0.91
	No	78	54	69.23%		74	38	51.35%		2.13 (1.09–4.16)	0.02
Occupation	Housewives	62	53	85.48%	< 0.001	71	43	60.56%	0.33	3.83 (1.65–9.32)	0.01
	Employee	16	7	43.75%		11	5	45.45%		0.93 (0.18–4.68)	0.94
	Student	12	4	33.33%		8	3	37.50%		0.83 (0.11–6.28)	0.86
Meat Consumption	Cooked	56	35	62.5%	0.02	67	34	50.75%	0.05	1.62 (0.78–3.36)	0.19
	Half Cooked	34	29	85.29%		23	17	73.91%		2.04 (0.51–8.23)	0.31
Education	None-Academic Educated	61	49	80.33%	0.005	69	44	63.77%	0.013	2.32 (1.04–5.28)	0.03
	Academic Educated	29	15	51.72%		29	15	51.72%		2.15 (0.66–7.12)	0.21
Awareness of *T. gondii*	Yes	16	5	31.25%	< 0.001	11	2	18.18%	< 0.001	2.04 (0.31–17.89)	0.49
	No	74	59	79.73		79	49	62.03%		2.41 (1.16–5.05)	0.01
Trimester of Pregnancy	Second	27	16	59.26%	0.12	39	21	53.85%	0.64	1.25 (0.46–3.44)	0.67
	Third	63	48	76.19%		51	30	58.82%		2.24 (0.99–5.06)	0.06
Miscarriage history	Yes	11	7	63.64%	0.57	9	4	44.44%	0.46	1.12 (0.18–6.91)	0.91
	No	79	57	72.15%		81	47	58.02%		1.87 (0.96–3.66)	0.06
Total	**-**	**90**	**64**	**71.11%**	**-**	**90**	**51**	**56.66%**	**-**	**1.88 (1.01–3.55)**	**0.045**

In the multivariable logistic regression, maternal age above 30y, housewives, none-academic educated, lack of *T. gondii* awareness, consumption of undercooked meat, and *T. gondii* IgG seropositivity were analyzed and the result indicated that being housewives and *T. gondii* IgG seropositivity are significant risk factors forPEp among pregnant women in this study population (*p* < 0.05, Table 2).

**Table 2:** Multivariable logistic regression analyses of the predictors for preeclampsia among pregnant women in Northern Iran

Variable	Coefficient	Standard Error	Odds Ratio	95% Confidence Interval	*p*-value
Maternal age > 30	0.75	0.52	2.11	0.76–5.81	0.14
Housewives	1.55	0.64	4.67	1.32–16.52	0.016
Consume meat (Half Cooked)	0.65	0.38	1.92	0.91–4.02	0.082
None Academic Educated	0.06	0.58	1.06	0.34–3.34	0.916
Lack of *T. gondii* awareness	0.05	0.67	1.05	0.27–3.87	0.938
*Toxoplasma* IgG seropositivity	1.01	0.37	2.75	1.34–5.67	0.005

## Discussion

PE remains a leading cause of maternal and perinatal morbidity and mortality, especially in low and middle-income countries, where limited access to early detection and care exacerbates outcomes [12, 20]. Despite affecting approximately 3% of pregnancies globally, [2]. In recent years, a growing body of evidence has linked infectious agents, particularly *T. gondii*, to hypertensive disorders of pregnancy.

Our findings revealed a significantly higher prevalence of anti-*T. gondii* IgG antibodies among preeclamptic women compared to normotensive controls with an OR of 1.88 (*P* = 0.045), suggesting a potential association between latent toxoplasmosis and increased PE risk. The relationship between *T. gondii* infection and PE exhibits significant epidemiological heterogeneity, reflecting complex interactions between parasitic factors, host immunity, and regional determinants. Serological studies reveal stark geographical discordance, while Sudanese women with PE demonstrate markedly higher anti-*T. gondii* IgG seropositivity (32.3% vs. 4.4%; *p* < 0.001) [21], Turkish [22] and Mexican [23] cohorts show no significant differences (27.3% vs. 20.5%; 6.2% vs. 5.5%, *p* > 0.05). This variability may arise from divergent parasite genotypes, host genetic polymorphisms in immune response pathways, or environmental cofactors influencing exposure dynamics.

Mechanistically, chronic toxoplasmosis potentiates PE pathogenesis through sustained maternal immune activation [24]. Latent infection maintains elevated pro-inflammatory cytokines (TNF-α, IL-6, IFN-γ) that disrupt placental angiogenesis by upregulating antiangiogenic factors like soluble fms-like tyrosine kinase-1 (sFlt-1), which induces endothelial dysfunction and systemic vasoconstriction [24–26]. Furthermore, *T. gondii* infection impairs trophoblast invasion by matrix metalloproteinase dysregulation and oxidative stress-mediated damage, exacerbating placental insufficiency [24, 25, 27]. These processes create a pro-thrombotic, antiangiogenic state mirroring the core pathophysiology of preeclampsia, suggesting that chronic infection may amplify preexisting maternal vulnerabilities rather than acting as a singular etiological agent.

Clinical and public health implications remain contentious due to methodological limitations in existing studies and contextual variability in seroprevalence. Although acute gestational infection unequivocally risks vertical transmission, its population-level contribution to preeclampsia is minimal given low seroconversion rates. Conversely, latent infection’s role is obscured by inconsistent seroepidemiological patterns and diagnostic challenges, particularly in interpreting IgG avidity tests [19]. Mitigating this burden requires multifaceted strategies: firstly, enhanced antenatal education targeting seronegative women especially critical in settings like northern Iran (Our study), where 82.22% of pregnant women exhibit poor toxoplasmosis knowledge despite high regional seroprevalence (71.11%). Secondly, standardized serological monitoring coupled with angiogenic biomarker profiling (e.g., sFlt-1/PlGF ratio) could identify high-risk pregnancies warranting immunomodulatory interventions [28].

Household activities, especially those involving contact with soil, cat feces, or raw meat, significantly increase the risk of *T. gondii* infection [29]. Similarly, gardening without gloves exposes people to soil contaminated with oocysts from cat feces, which can survive for more than a year in warm, moist environments. Food preparation tasks also pose risks: handling undercooked meat can introduce tissue cysts or oocysts into the digestive tract [29]. For pregnant women, these activities are of particular concern, as acute infection can lead to congenital toxoplasmosis associated with miscarriage, neurological damage, or eye damage. On the other hand, household physical activities and psychosocial stress may contribute to the pathogenesis of preeclampsia through hemodynamic and inflammatory pathways. Strenuous activities (Such as prolonged standing, heavy lifting, or vigorous cleaning) can cause physiological stress and reduce uteroplacental perfusion by diverting blood flow to the working muscles. This ischemia-reperfusion injury in the placenta increases the release of antiangiogenic factors (Such as soluble fms-like tyrosine kinase-1, sFlt-1) and causes maternal endothelial dysfunction as a hallmark of PE [30]. Epidemiological studies report a 3.1-fold increased risk of preeclampsia in pregnant women with high-stress jobs, a finding that could be extended to strenuous housework, particularly in resource-limited settings where the household burden is often not shared [31]. Physical strain may also exacerbate oxidative stress and inflammatory cytokine production (Such as TNF-α), further disrupting the angiogenic balance and increasing preeclampsia-related organ damage [32].

Lower education levels may be a key socioeconomic factor driving *Toxoplasma* infection rates, likely due to limited healthcare access, health education, and preventive measures [33]. The study found that women with university education had lower seroprevalence than less-educated women, attributed to greater health literacy and awareness of preventive practices such as proper food handling and hygiene. Women with lower education levels may lack knowledge about *Toxoplasma* prevention including risks of undercooked meat and hygiene practices, leading to higher exposure and infection rates. They may also face socioeconomic challenges, including poorer living conditions and limited access to preventive resources. Similar to Eroglu et al., [34], who linked higher *T. gondii* rates in less-educated women to unsafe dietary practices and poor hygiene [34], this study associates higher education with lower infection prevalence due to increased preventive behaviors. Thus, the study underscores education’s pivotal role in mitigating *T. gondii* risk in pregnant women. Lower education levels (Particularly high school education or below) correlated with higher infection rates, likely due to limited health literacy and preventive resources. Public health strategies targeting health education especially for less-educated women could reduce *T. gondii* infections and related complications like PE.

Overall, the findings highlight the role of health education in preventing *T. gondii* infection. Women unaware of the disease are more likely to engage in high-risk behaviors (e.g., consuming undercooked meat or poor pet hygiene), increasing infection likelihood. Conversely, informed women are more likely to adopt preventive measures (e.g., avoiding undercooked meat, washing produce, and maintaining hygiene), significantly reducing risk. Comparisons with other studies support these findings. Eroglu et al., [34] showed that increased toxoplasmosis awareness reduced infection rates, particularly in pregnant women [34]. Ait Hamou et al., [35] and Maartens et al., [36] also noted preventive education for high-risk populations. Thus, this study emphasizes the need to raise *T. gondii* awareness among pregnant women, especially high-risk groups like those with PE. Health education campaigns could reduce infections and potentially lower pregnancy complications like preeclampsia. Public health initiatives should prioritize preventive education to improve maternal outcomes.

In present study, consumption of undercooked meat was significantly associated with higher *T. gondii* infection rates, particularly among women with preeclampsia (85.29% vs. 56.25%). This reinforces the importance of thorough meat cooking to prevent *Toxoplasma* infection, especially in pregnant women, to reduce complications like PE. Although no significant dietary differences were observed between preeclamptic and healthy pregnant women, larger studies with refined controls are needed to examine the roles of dietary habits, immune responses, and environmental factors in PE pathogenesis. Enhanced food safety education is also critical for minimizing infection risks in pregnant women [37].

Our study demonstrates a significant association between toxoplasmosis and PE in women aged > 30 years. On one hand, toxoplasmosis becomes more common by getting older primarily due to cumulative exposure to *T. gondii* through lifelong environmental and dietary routes leading to seroprevalence rates that increase [38]. On the other hand, PE rises in incidence after age 30, largely because advanced maternal age exacerbates underlying vascular and metabolic vulnerabilities such as chronic hypertension, diabetes, and obesity [39]. Both conditions thus reflect intertwined effects of age-dependent immune decline and accumulated physiological stressors.

This study has several strengths, including its matched case-control design, robust serological testing, and multivariate analysis of behavioral and sociodemographic factors. However, limitations include the relatively small sample size, lack of longitudinal data, and inability to confirm active infection through PCR or other molecular diagnostics. Furthermore, causality cannot be established, and residual confounding may persist despite statistical adjustments.

## Conclusion

This study demonstrates a significant association between latent toxoplasmosis and an increased risk of PE, likely due to a complex interaction of host immune responses and environmental factors. Biomechanical and psychosocial stressors associated with housework have a greater impact on both toxoplasmosis and the pathogenesis of PE, suggesting that integrating these factors into prenatal counseling may reduce adverse outcomes. Lower educational levels and lack of awareness of *T. gondii* among pregnant women are associated with higher rates of infection, highlighting the role of education as a key modifiable factor in infection control. The findings suggest that undercooked meat consumption significantly increases the risk of infection, reinforcing the need for targeted food safety education. Furthermore, increasing maternal age contributes to cumulative exposure to *T. gondii* and is associated with an increased incidence of PE due to age-related physiological vulnerabilities, suggesting overlapping risk pathways. Taken together, these insights support comprehensive public health strategies prioritizing educational interventions and structural considerations to reduce *T*. *gondii *infection and its contribution to PE, thereby improving maternal and newborn health outcomes globally.

## Data Availability

The datasets used and/or analysed during the current study are available from the corresponding author on reasonable request.

## Abbreviations

PE: Preeclampsia
ELISA: Enzyme-Linked Immunosorbent Assay
